# Supplementary material for: Polyoxymethylene as Material for Removable Partial Dentures—A Literature Review and Illustrating Case Report
Source: J Clin Med. 2021 Apr 2;10(7):1458. doi: 10.3390/jcm10071458 (PMC8038064; doi:10.3390/jcm10071458)
Supplement: Supplementary file 1 [file jcm-10-01458-s001.pdf]

Supplementary Materials

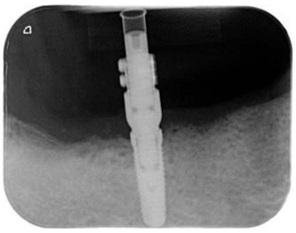

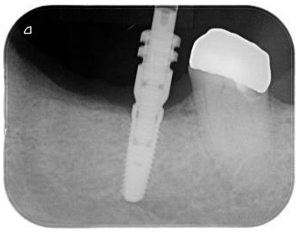

(a) **(b)**

Figure S1. X-ray of the implants set into the lower jaw with impression posts, (**a**) right molar area (**b**) left molar area.

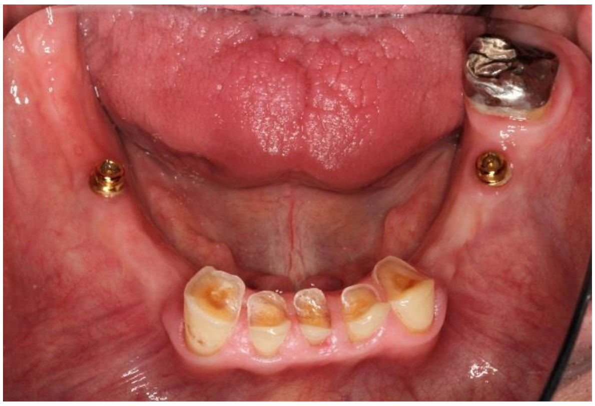

Figure S2. Occlusal view of the lower jaw after implant insertion and locator abutments.
